# Predictive Models of Phytosterol Degradation in Rapeseeds Stored in Bulk Based on Artificial Neural Networks and Response Surface Regression

**DOI:** 10.3390/molecules27082445

**Published:** 2022-04-10

**Authors:** Jolanta Wawrzyniak, Magdalena Rudzińska, Marzena Gawrysiak-Witulska, Krzysztof Przybył

**Affiliations:** Faculty of Food Science and Nutrition, Poznań University of Life Sciences, 60-624 Poznań, Poland; magdalena.rudzinska@up.poznan.pl (M.R.); marzena.gawrysiak-witulska@up.poznan.pl (M.G.-W.); krzysztof.przybyl@up.poznan.pl (K.P.)

**Keywords:** phytosterol degradation, rapeseed storage, artificial neural networks, response surface regression, predictive modeling, postharvest preservation systems

## Abstract

The need to maintain the highest possible levels of bioactive components contained in raw materials requires the elaboration of tools supporting their processing operations, starting from the first stages of the food production chain. In this study, artificial neural networks (ANNs) and response surface regression (RSR) were used to develop models of phytosterol degradation in bulks of rapeseed stored under various temperatures and water activity conditions (*T* = 12–30 °C and *a_w_* = 0.75–0.90). Among ANNs, networks based on a multilayer perceptron (MLP) and a radial basis function (RBF) were tested. The model input constituted *a_w_*, temperature and storage time, whilst the model output was the phytosterol level in seeds. The ANN-based modeling turned out to be more effective in estimating phytosterol levels than the RSR, while MLP-ANNs proved to be more satisfactory than RBF-ANNs. The approximation quality of the ANNs models depended on the number of neurons and the type of activation functions in the hidden layer. The best model was provided by the MLP-ANN containing nine neurons in the hidden layer equipped with the logistic activation function. The model performance evaluation showed its high prediction accuracy and generalization capability (*R*^2^ = 0.978; *RMSE* = 0.140). Its accuracy was also confirmed by the elliptical joint confidence region (EJCR) test. The results show the high usefulness of ANNs in predictive modeling of phytosterol degradation in rapeseeds. The elaborated MLP-ANN model may be used as a support tool in modern postharvest management systems.

## 1. Introduction

Phytosterols are compounds found naturally in plants that have nutritional value and a proven medicinal effect on the human body. Their bioactive properties result from the fact that these plant sterols, both in terms of functional properties and chemical structure, closely resemble cholesterol [1]. In plant cells, phytosterols, being major components of phospholipid bilayer membranes, play a stabilizing role similarly to cholesterol in animal cell membranes [2]. In turn, slight differences in the chemical structure of phytosterol compared to the cholesterol molecule result only from an extra methyl or ethyl group and/or a double bond at the C-5 position of the ring and 1–2 more atoms in the side chain. As a consequence of the similarity in the chemical structures of both above-mentioned molecules, phytosterols are able to inhibit the absorption of cholesterol from the digestive tract into the blood in the circulatory system [3,4]. In nature, more than 250 sterols and their derivatives have been identified [5]. They can occur as free sterols or as four related compounds, in which the 3-hydroxyl group is esterified to a fatty acid or hydroxycinnamic acid, or glycosylated with a hexose (usually glucose) or a 6-fatty acyl hexose [6]. The most predominant phytosterols present in plant foods include campesterol, β-sitosterol, stigmasterol and brassicasterol [1,6,7]. Due to the bioactive properties and therapeutic potential of phytosterols, in recent years, growing interest in their use in the food industry has been observed.

In the human diet, phytosterols are supplied mainly as components of plant oils, in which they constitute the unsaponifiable fraction [8]. Previous research proved that only intake of phytosterols at a level higher than 1.5 g day^−1^ leads to lower plasma LDL-cholesterol concentrations [4]. As a diet composed of natural sources is able to supply these bioactive molecules at 0.2–0.4 g day^−1^, in recent years numerous attempts have been made to develop new phytosterol-enriched products, such as margarine or dairy foods (milk, yogurt and yogurt drinks) [9,10,11].

Among vegetable oils rapeseed oil is one of the richest sources of phytosterols with the concentration varying between 5–10 g kg^−1^ of oil [7,12,13,14] depending on the genetic background, planting location and environmental conditions during flowering and seed ripening [12,14]. The amount of these bioactive molecules can be significantly reduced during the first stages of the food production chain [12]. The reason for the drop in phytosterol levels is presumably connected with their chemical activity resulting from the presence of double bonds in their molecule. As a consequence, these components can easily undergo undesirable processes, promoting the breakdown of lipids, especially unsaturated ones. In turn, their breakdown products, such as aldehydes, may lead to further damage to proteins and nucleic acids [2,15]. In view of the above, great attention should be paid to rapeseed postharvest treatments, as the omission or inadequately performed postharvest preservation may intensify those processes and lead to a deterioration of the seed’s technological quality, including the lowering of the phytosterol content.

The research on the impact of temperature on the phytosterol degradation rate in rapeseeds has shown that high-temperature drying (60–120 °C) causes a more rapid loss of phytosterols compared to near-ambient drying [16,17,18]. The milder drying conditions used in near-ambient drying in a thick layer promote greater retention of these bioactive components in the raw material but contribute to an extension of the processing time. For most of this drying time, moisture content (MC) in the upper layer of seed bulk remains at the initial level, often exceeding the value of 7–8% w.b. (wet basis) suggested for long-term safe storage of rapeseed [19,20,21]. Additionally, in the mass of seeds located in the northern (cooler) part of a silo, moisture spots may appear, which may be caused by the transfer of moisture from seeds placed in the southern part of the silo, being warmer, heated by the sun. Elevated moisture content persisting over a longer period of time and moisture migration in the mass of seeds may promote mold growth and self-heating processes. These undesirable phenomena in bulk stored rapeseeds may intensify the oxidation of native biological compounds, such as fatty acids or phytosterols, leading to the formation of toxic products, e.g., free radicals, lipid peroxides, aldehydes, ketones, etc. [15]. A reduction in the content of phytosterols and tocopherols in rapeseeds stored under adverse conditions, typically accompanied by an increase in acid value and a decrease in seed germination, has been confirmed in previous studies [7,22].

The main concerns in the edible oil industry are connected with maintaining the highest possible quality of seeds and minimizing the loss of bioactive compounds contained in them, such as phytosterols. To retain bioactive compounds and thus sustain the high nutritional quality of rapeseed oil, postharvest process management systems are being constantly modernized and upgraded. Tools facilitating the prediction of the direction of changes in the quality of the preserved raw material can play a significant role in the improvement of the above-mentioned systems, but they need to be based on data gathered in systems as closely resembling actual ones as possible. Our previous study focused on identifying conditions (temperature, water activity in seeds and time of storage) conducive to phytosterol degradation in the bulk of stored rapeseeds [7]. Using these data, in this study, an attempt was made to develop a model based on artificial neural networks (ANNs) and response surface regression (RSR) to predict the kinetics of phytosterol degradation in a stored bulk of rapeseeds with a high level of fungal propagules typical of regions and years with heavy rainfall during seed ripening and harvest.

## 2. Results

The phytosterol content in rapeseed oil depends on the quality of the seeds used in its production. It was proven that phytosterol degradation is often associated with changes in the acid value (AV) and germination of the seeds [7,17,22,23]. Nevertheless, a previous study has shown that the kinetics of changes in PS, AV and seed germination differ significantly [7]. For this reason, AV and seed germination, whose undoubted advantage is the ease of their determination, cannot be used as indicators of phytosterol decomposition processes. This is due to the fact that it is not feasible to estimate a single universal limit of AV and seed germination, indicating the risk of a significant reduction in phytosterol content in stored rapeseeds. Since the determination of the phytosterol content requires some research equipment and knowledge, it is worth considering the use of modeling techniques to estimate the level of these compounds in seeds. Mathematical modeling is an increasingly common method used in food technology and agriculture. Predictive models support food processes by forecasting the microbiological quality and physicochemical properties of raw materials and food products [24,25,26,27,28,29,30,31]. In this study, an attempt was made to develop a model of phytosterol degradation in rapeseeds stored under a wide range of conditions using artificial neural networks and response surface regression.

### 2.1. Artificial Neural Network Model

**Figure 1:**
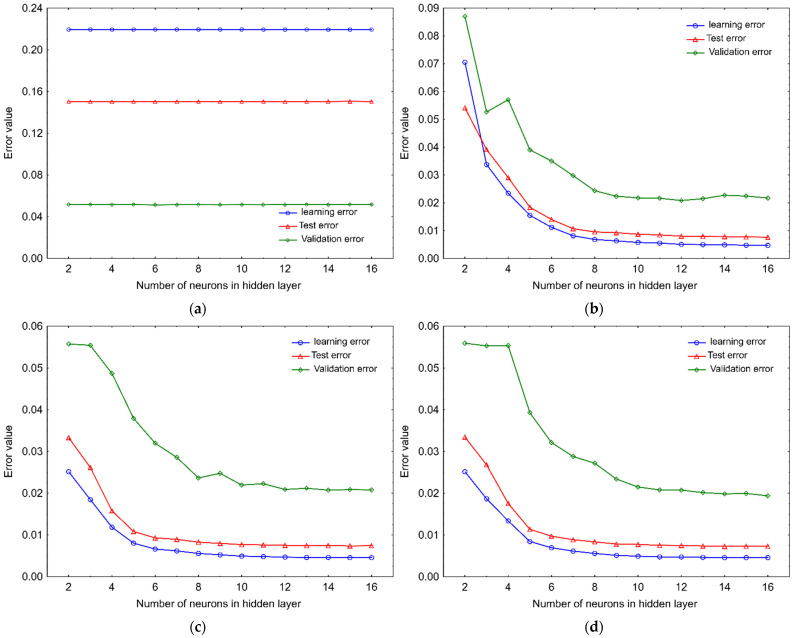
Changes in mean values of training, test and validation errors of MLP networks applied to modeling of phytosterol degradation depending on the number of neurons in the hidden layer equipped with an activation function in form of (**a**) a linear, (**b**) an exponential, (**c**) a hyperbolic tangent and (**d**) a logistic function.

ANNs with various architectures have previously been applied for the prediction of the quantity of such bioactive compounds as phenolic, essential oils and polyunsaturated fatty acids [32,33,34,35,36,37]. In this study, ANNs were analyzed in terms of their ability to predict the dynamics of phytosterols degradation in a bulk of rapeseeds stored in a wide range of conditions encountered in agricultural practice. The development of the neural network model included the exploration of 60 MLP-based and 50 RBF-based network topologies. All the examined networks consisted of three layers, i.e., the input, single hidden and output layer. The input layer involved three neurons corresponding to the independent variables (temperature (*T*) and water activity in seeds (*a_w_*), and the time of storage (*τ*)), whilst the output layer was formed of a single neuron corresponding to the dependent variable. Contrary to the input and output layers, which configuration directly depends on the data nature, the design of the structure of the hidden layers is much more complex and requires much more attention [38]. There are several practical rules in the literature that can support the design of network structures and allow for a preliminary estimation of the number of neurons in the hidden layer [39,40,41,42]. In the study, one of those formulas assuming five learning cases for each model coefficient [25,42] was also used for a preliminary estimation of the number of neurons in the hidden layer of the MLP-ANNs. Once the number of neurons is initially determined, a more extensive trial and error method of the effect of structure on results is usually performed [38,43]. The networks tested in this study contained a single hidden layer with different numbers of neurons, i.e., from 2 to 16 and from 2 to 50 for MLP-ANNs and RBF-ANNs, respectively. Our investigations proved that neural networks are an effective tool for modeling the level of phytosterol in a bulk of rapeseeds. The obtained results showed that the most vital features for network performance are the number of neurons and the type of activation function in the hidden layer. The analyzes of parameters describing the quality of the constructed MLP-based topologies, i.e., the learning, test and validation error, revealed that structures containing the linear activation function in the neurons of the hidden layer were not successful in predicting the PS level in a stored mass of rapeseeds. For these networks, the mean values of the above-mentioned errors remained at constant and simultaneously relatively high levels, regardless of the number of neurons in the hidden layer (Figure 1a), therefore they were excluded from further deliberation. In the case of other tested MLP-based structures, the mean values of learning and test errors initially significantly decreased with an increase in the size of the hidden layer, then for topologies containing more than 8 neurons, they stabilized and remained more or less at the same level of approx. 0.0046–0.0047 and 0.0073–0.0077, respectively (Figure 1b–d). One of the most important measures of network quality is the validation error, which describes the accuracy of its response to new data and its ability to generalize. Changes in this error had a similar course in relation to the two previous ones, except that in the case of the structures containing Than and Log in neurons of the hidden layer, its noticeable decrease begun when the number of neurons in the hidden layer exceeded 3 and 4, respectively. Finally, the value of the validation error settled down at the level of approximately 0.020 for structures that contained 10 or more hidden neurons.

It is known, that overly complex topologies tend to remember individual cases and lose the generalization ability, which is known as overfitting [44]. The analysis of the MLP-ANN quality showed that networks with about 10 neurons in the hidden layer are the most balanced in terms of simplicity and prediction efficiency (Figure 1). These topologies are characterized by relatively high-quality metrics and quite compact structures that can ensure their resistance to overfitting. The result of these deliberations was consistent with the number of nodes in the hidden layer estimated by the mentioned earlier rule of thumb formula, assuming five learning cases for each coefficient of the model of the network (9–10 nodes). Structures with similar topology have been used in previous studies to estimate the yield of bioactive components or levels of microbes in raw material. The MLP-ANN topology containing a comparable number of nodes in a single hidden layer (5-8-1) was adopted as the optimal model for predicting the eugenol content in extracts obtained from basil leaves [34]. The MLP-ANN model with the 4-10-1 topology was considered the best for estimating the levels of chlorogenic acid in extracts from *Lonicera japonica* [33]. In turn, a multilayer perceptron with a single hidden layer containing five nodes was selected as the best architecture for the prediction of the fungal population in an ecosystem of stored barley grain [25].

**Figure 2:**
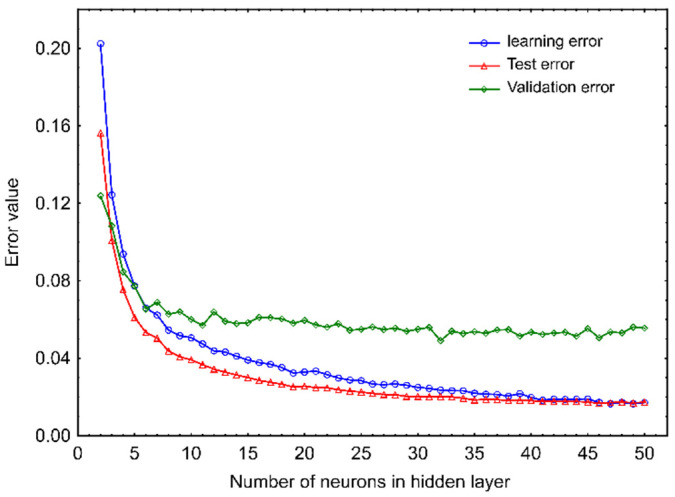
Changes in mean values of training, test and validation errors of RBF networks applied to modeling of phytosterol degradation depending on the number of neurons in the hidden layer equipped with an activation function in form of a gaussian function.

Consideration conducted for the RBF-ANNs showed that an increase in the number of neurons in the hidden layer from 2 to 20 also caused a significant drop in the mean values of learning and test errors (Figure 2). Further increases in the number of hidden neurons to 50 resulted in a much milder reduction in their values, which finally reached the same level of approximately 0.017. The average value of the validation error for the aforementioned networks stabilized much earlier than those of the learning and test errors; as for structures containing more than 10 hidden neurons, it was close to the value of about 0.05. The lowest level of the mean value of the validation error was recorded for the network with 32 nodes in the hidden layer (0.049). The above-described results obtained for RBF-ANN allow us to conclude that networks with 20–40 hidden neurons constitute the best compromise between the size of the network and the efficiency of prediction of phytosterol degradation in a stored bulk of rapeseeds.

It is worth mentioning, that the typical error curves initially decrease to a certain level as the number of nodes in the hidden layer increases, then, with further enlargement of the hidden layer, the training error usually tends to continue to decrease, while the validation error value may begin to increase, indicating the network overfitting associated with remembering individual cases and the loss of its ability to generalize. This tendency was also observed in our previous studies on predictive models for the assessment of the mycological state of barley and rapeseed ecosystems [25,45]. Nevertheless, when building ANNs, one can encounter a number of other common situations [38]. In this research, the validation errors after the initial decline stabilized at an almost constant level and did not show an upward trend. Similar trends in validation error curves were observed by Mateo et al. [46] investigating the ability of MLP neural networks and RBF networks to predict the ochratoxin A (OTA) in grape-based cultures of *Aspergillus carbonarius*. As reported by Basheer and Hajmeer [38], such a situation means that further enlargement of the network structure does not improve its quality and if the error values are acceptable, the final network architecture should be looked for in the area of this steady behavior. Since all error values obtained in the study were satisfactory, further considerations were focused on the rational selection of a network, which could be adopted as a model for phytosterol degradation.

Out of all the tested topologies, two networks based on different ANN types (MLP and RBF) and characterized by the best predictive efficiency were selected as neural network models for the phytosterol degradation in bulk stored rapeseed. The best network topology was evaluated on the basis of the lowest weighted average of learning, test and validation errors (where the share of individual errors was proportional to the size of the corresponding data set and accounted for 52, 23 and 25%, respectively). The structure and quality metrics of the selected MPL and RBF neural network models are presented in Table 1. The best MLP-ANN model predicting phytosterol degradation was a network with 9 neurons in the hidden layer equipped with a Log transformation function, whereas the best RBF-ANN model contained 27 neurons in the hidden layer. The accuracy of the RBF-ANN model was slightly lower than that of the MLP-ANN model, but its prediction quality was still satisfactory. Previous studies on the ability of MLP-ANN and RBF-ANN to predict the accumulation of deoxynivalenol (DON) in barley grain contaminated with *Fusarium culmorum* under various conditions have shown that both MLP-ANNs and RBF-ANNs give the possibility of accurately predicting DON levels; nevertheless, RBF-ANNs require more nodes in the hidden layer to achieve performance similar to that of the MLP-based networks [47]. Studies on the utility of artificial neural networks for predicting fungal populations in the ecosystem of stored rapeseeds also showed that the predictive quality of the best-acting MLP-ANN (3-12-1) and RBF-ANN (3-30-1) networks was not significantly different [45]. In this research, the RBF network exhibited a slightly inferior performance than MLP-ANN, although it contained a much larger number of neurons in the hidden layer.

### 2.2. Response Surface Regression Model

Second-order surface response regression (RSR) was also used to develop a model to predict phytosterol degradation in a stored bulk of rapeseeds. The RSR model was formulated as a function of three independent variables (*a_w_*, *T*, *τ*). The values of model coefficients determined during the regression analysis are presented in Table 2. The obtained results revealed that regression coefficients of all the considered equation factors, i.e., the first and second power and the two-way interaction of each predictor variable, were statistically significant (*p* < 0.05). Therefore, all the factors included in the general form of the equation were included in the model.

As a result, the RSR model took the following form:

(1) $$y=\beta_{0}+\beta_{1}\cdot a_{w}+\beta_{2}\cdot a_{w}^{2}+\beta_{3}\cdot T+\beta_{4}\cdot T^{2}+\beta_{5}\cdot \tau +\beta_{6}\cdot \tau^{2}+\beta_{7}\cdot a_{w}\cdot T+\beta_{8}\cdot a_{w}\cdot \tau +\beta_{9}\cdot T\cdot \tau =-55.1677+132.8619\cdot a_{w}-69.4989\cdot a_{w}^{2}+0.9326\cdot T-0.0037\cdot T^{2}+0.2277\cdot \tau +0.0001\cdot \tau^{2}-1.0046\cdot a_{w}\cdot T-0.2870\cdot a_{w}\cdot \tau -0.0013\cdot T\cdot \tau$$

**Figure 3:**
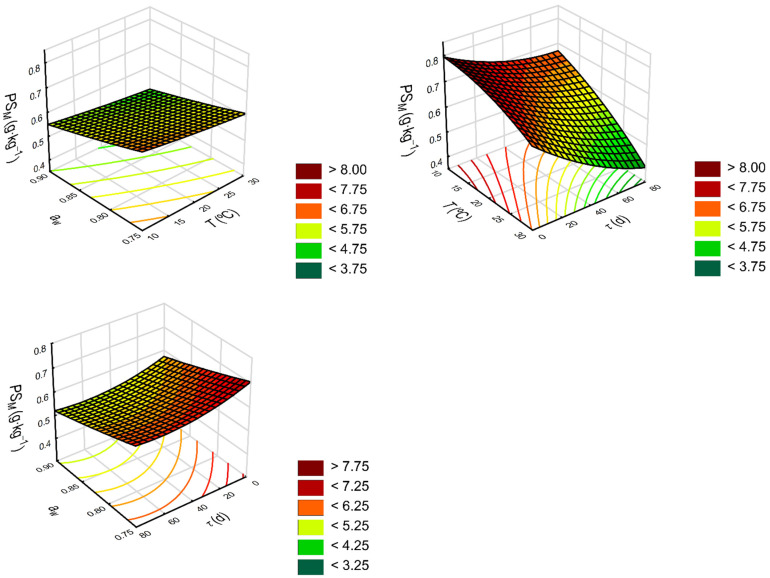
Response surface presenting the effects of temperature (*T*), water activity in seeds (*a_w_*) and storage time (*τ*) on phytosterol concentration (*PS_M_*).

The model responses showed fairly decent agreement with the experimental data used in model building (*R* = 0.959). The response surfaces for phytosterol concentration depending on storage temperature, water activity in seeds and time are visualized in Figure 3. Satisfactory results for the quadratic model developed with the use of response surface regression were also obtained by Santos et al. [48] when estimating the total phenolics in rose petals intended among others for cooking (*R* = 0.984).

### 2.3. Model Performance Evaluation

**Figure 4:**
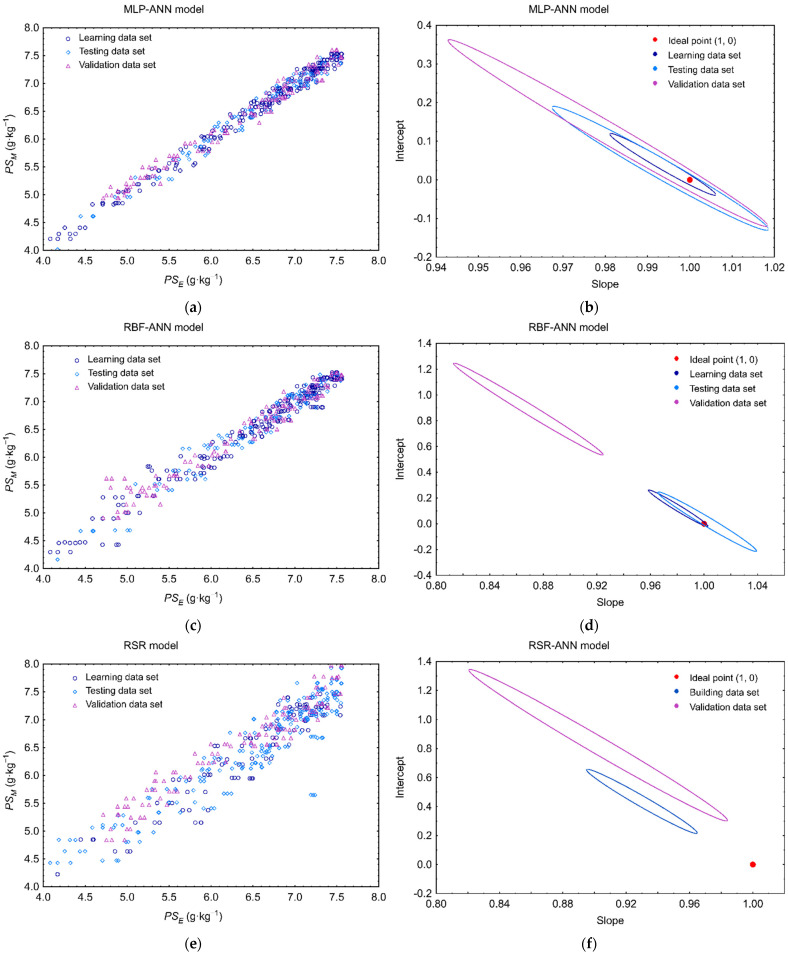
Predictability of (**a**) MLP-ANN (**c**) RBF-ANN and (**e**) RSR model of phytosterol degradation in a stored bulk of rapeseed (*PS_M_*) in relation to the experimental values of phytosterol content (*PS_E_*) adopted from Wawrzyniak et al. [7], together with elliptical joint confidence regions at a 95% level for the slope and intercept of the regression of predicted *PS_M_* versus experimental *PS_E_* using ordinary least squares, respectively, for (**b**) MLP-ANN, (**d**) RBF-ANN, and (**f**) RSR model.

The prognostic efficiency and the usefulness for the practical applications of all elaborated models of phytosterol degradation were assessed using statistical indices recommended for predictive model validation [49,50]. The model performance evaluation was carried out based on data used to model developments (internal validation), and additionally, on the experimental data set (a validation data set) collected in four independent experiments (external validation). The predicted values of phytosterol content plotted against the observed data (Figure 4) showed good agreement between the experimental and predicted points, and were confirmed by high values of correlation coefficients (*R_MLP-ANN_* = 0.9887, *R_RBF-ANN_* = 0.9769, *R_RSR_* = 0.9661 for the validation data set).

To take a closer look at the predictive effectiveness of the designed models, they were subjected to a more comprehensive statistical assessment employing indicators that are commonly used to evaluate prognostic models. Detailed results of the statistical evaluation for the performance of each elaborated model are summarized in Table 3. The verification of the models based on the determination coefficient (*R*^2^), root mean square error (*RMSE*) and mean absolute error (*MAE*) showed that all of them were characterized by a relatively high goodness-of-fit to the experimental data. The best predictive ability was noted for the MLP-ANN model, which was distinguished by a high goodness-of-fit to new experimental data (*R*^2^) and the lowest value of *RMSE* and *MAE*. The RBF-ANN and RSR were characterized by a slightly lower accuracy in predicting phytosterol degradation in a stored bulk of rapeseed than the MLP-ANN. The values of the mean relative percentage error (*MRPE*, %) indicates that the mean deviation of the model response from the experimental data in the case of MLP-ANN did not exceed 1%. For the RBF-ANN and RSR models, they reached the levels of 1.2% and 3.5%, respectively. The value of the bias factor (*B_f_*) close to unity suggests that positive and negative deviations of the model are approximately equal [49]. The study reported values of *B_f_* slightly higher than 1 and negative values of *MRPE* (except for their values determined for test data sets in the case of ANN models), which indicates that approximations of developed models can slightly overestimate predicted phytosterol levels [49,50]. Nevertheless, such low values of mean deviations, in particular for the MLP-ANN network, should not have a significant impact on the responses returned by the models.

As counter-directional deviations can cancel each other when calculating *MRPE* and *B_f_*, the overall distance between the approximated and observed levels of phytosterol contents was also determined with the use of the mean absolute relative percentage error (*MAPE*, %) and the accuracy factor (*A_f_*). Both these indices show that an overall prediction error of the ANN models amounts to approx. 2–2.5%, while for the model formulated with RSR, it was over 4%.

The obtained results showed that artificial neural networks proved to be more effective in the estimation of the examined bioactive components than the RSR method. Similar results were observed by [51] Carbone (2020) when predicting natural antioxidant content in an extract from kiwi fruit pomace. They observed that the ANN model exhibited more accurate predictions and better generalization than the model based on the response surface methodology (RSM), (*R*^2^: 0.90 and 0.99 for RSM and ANN, respectively). A comparison of the response surface methodology and artificial neural network modeling, in terms of their application to assess glycosides and total phenolics content in extracts of *Stevia rebaudiana* (Bertoni) leaves, showed that the ANN models are an attractive alternative to the RSM due to their better estimation and prediction capabilities. In turn, Hui-Chuan et al. [33] found the superiority of ANN over the quadratic response surface model in predicting the extraction efficiency of the chlorogenic acid (CGA)-a bioactive compound commonly found in plants. Comparable results showing that ANN models are quite better at forecasting biomolecule quantities than models based on the RSM methodology have also been found by other researchers [32,37,52,53].

Overall, the study shows the high usefulness of the ANN-based modeling technique in predicting the phytosterol degradation in a stored bulk of rapeseeds. However, in our case, MLP-based networks had a slight advantage in operating efficiency over RBF networks. It is consistent with the previous studies. The greater suitability of MLP-ANNs in phenolic compounds quantification has been reported by Torrecilla et al. [54]. Mateo et al. [46], using networks based on RBF and MLP to predict the accumulation of ochratoxin A in grape juice as a function of the *a_w_*, temperature and carbendazim fungicide doses, also observed that the overall performance of RBF-based networks was inferior to MLP-ANNs. A study comparing the use of RBF-ANNs and MLP-ANNs with a single hidden layer to predict fungal infestation in rapeseed ecosystems stored under various *a_w_* and temperatures also revealed a slight superiority of MLP-ANNs over RBF-ANNs [45].

**Figure 5:**
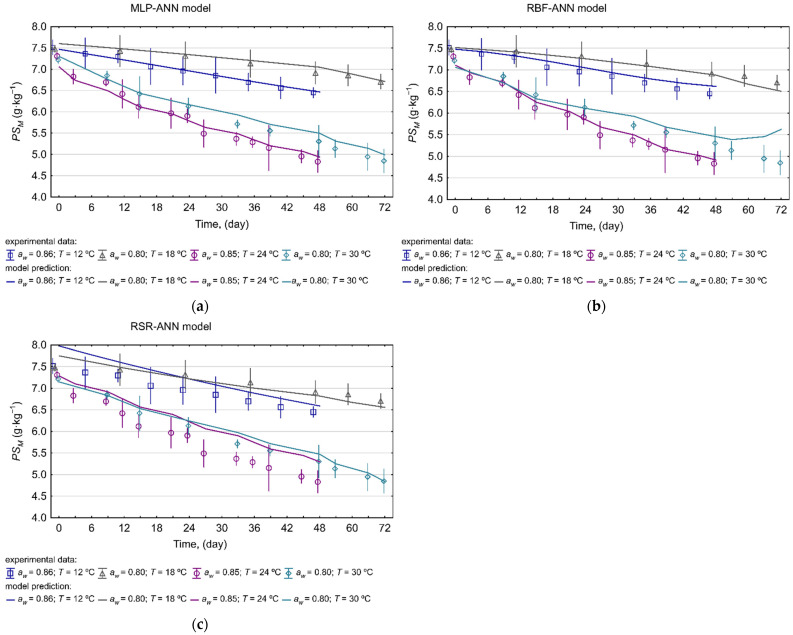
The comparison of (**a**) MLP-ANN, (**b**) RBF-ANN, (**c**) RSR model predictions (*PS_M_*, lines) and observed phytosterol levels (*PS_E_*, points) in rapeseed ecosystems adopted from Wawrzyniak et al. [7] for validation data set.

The elliptical joint confidence region (EJCR) test is a helpful method often used to assess the accuracy of fitting reference values to predicted ones [55,56,57,58,59]. The EJCR was also used to evaluate the convergence of the model predictions and experimentally determined the PS content. During this trial, the intercept and the slope of the correlation curve between the predictied and observed data were determined for each model, and parameters of these relationships were compared with the ideal values (1, 0). The EJCR confirmed that the MLP-ANN model could allow for accurate determination of the PS content in rapeseeds, as the confidence regions created for each individual data set contain the theoretically expected values (1, 0) (Figure 4), which shows that the experimental values of the PS content and the model responses are not significantly different at the 95% confidence level. In the case of the RBF-ANN model, the ideal values (1, 0) are included in the elliptical confidence region created only for training and test data sets, indicating that there is a bias between the model responses and validation data set. The elliptical confidence regions recorded for the RSR model indicate a greater dispersion between the experimental and predicted values of PS content for both the build and validation data sets, and they do not include the ideal values of intercept and slope. The outcomes of the EJCR test are consistent with the previously performed assessment of the predictive quality of the models based on the values of statistical factors, indicating the MLP-ANN model as the most accurate in approximating the phytosterol levels in stored rapeseed ecosystems. The higher performance of the MLP-ANN model over the other two is also evident in the plots when comparing the MLP-ANN, RBF-ANN and RSR model forecasts (*PS_M_*) with the observed phytosterol levels (*PS_E_*) in stored rapeseed ecosystems for the validation data set (Figure 5).

Considering the fact that phytosterol degradation may result in the formation of oxidized derivatives posing a risk of harmful effects on the human body [60], the use of the developed MLP-ANN model as a supporting predictive tool in postharvest management systems can be very helpful in preventing the hazards associated with the consumption of sterol oxidation products. The fact that the model of phytosterol degradation has been elaborated on the basis of data collected in experiments reflecting a bulk of seeds with a hazardous initial level of fungal spores, and that it predicts the level of phytosterol content in time as a function of seed temperature and water activity that are readily measurable in practice parameters, additionally increases its application value.

## 3. Materials and Methods

### 3.1. Experimental Data Collection

Data provided by Wawrzyniak et al. [7], describing changes in phytosterol contents in bulk stored rapeseeds with an adverse initial level of mold propagules, characteristic of seed ripening and/or harvested under unfavorable weather conditions, were used to model the kinetics of phytosterol degradation. The modeled data were collected in sixteen storage experiments, reflecting conditions (temperature *T* = 12–30 °C and water activity *a_w_* = 0.75–0.90) typical of regions with climates being a mixture of temperate maritime and continental, where most of the rapeseed production in the world takes place (approx. 96%), including, among others, Western and Central Europe and agricultural areas on the border between the United States and Canada (http://www.fao.org, accessed on 1 February 2022). For the transparency of this work and easier understanding of the research, in which the modeled data were collected, a short description of the experiment methodology is presented below.

#### Rapeseed Preparation and Experimental Design

Before the experiments, the rapeseed with moisture content on a wet basis (MC_w_._b_.) of 6.89 ± 0.23%, contaminated with fungal propagules at 3.1 ± 1.0 × 10^4^ CFU g^−1^ (the number of colony-forming units of molds per g of seeds) was adjusted to the assumed storage conditions according to the procedure described in a previous study [23]. For this purpose, seed samples of 4 kg were moistened to an MC_w_._b_. corresponding to the relative humidity (RH) at the equilibrium state in seed-intergranular spaces (ERH, where ERH = 100·*a_w_*, %) at a given temperature. The adequate level of an MC_w_._b_ was calculated using Halsey’s equation [61]. Then, the wetted seeds, infested with natural mycobiota, were additionally inoculated according to the procedure described by Wawrzyniak et al. [23,62] with spores of two toxigenic, fungal strains common in temperate climate regions, i.e., *Aspergillus ochraceus* Wilhelm (KKP 439) and *Penicillium verrucosum* Westling, (KKP 480) obtained from the Collection of Industrial Microorganisms (IAFB 212), the Institute of Agricultural and Food Biotechnology in Warsaw (Poland). The total level of fungal propagules at the beginning of the experiments was 4.5 -5.0 × 10^4^ CFU g^−1^ of seeds and it reflected the natural fungal populations in ecosystems of rapeseeds ripening and harvested in years with precipitation levels [63]. Afterwards, seed samples were placed in an environmental chamber described previously by Wawrzyniak et al. [62] to maintain steady humidity and temperature conditions (Table 4). Seeds were stored for 72 days in the case of *a_w_* = 0.75–0.81 and 48 days in the case of *a_w_* = 0.86–0.90.

The assumed levels of water activity were kept by maintaining the RH in the seed-intergranular spaces at a constant level corresponding to the assumed ERH using saturated salt solutions (NaCl, KBr, KCl, NH_4_Cl, BaCl_2_, Sr(NO_3_)_2_). Temperature and ERH were monitored throughout all the experiments that were conducted in two repetitions for each set of temperatures and *a_w_* in seeds. During these long-term storage experiments, the changes in phytosterol content were examined (in triplicate) using the Folch [64] and the AOCS [65] methods for oil extraction and saponification according to the procedure previously described by Wawrzyniak et al. [7].

### 3.2. Modeling Process

#### 3.2.1. Data Sets

The data on the phytosterol content in rapeseeds (PS, mg g^−1^) collected during storage experiments (Table 4) were used in further studies to model their degradation kinetics as a function of temperature, water activity and time. The data—recorded in twelve experiments covering a wide range of environmental conditions—were used to construct a model (building data set). In the case of the artificial neural network before the modeling process, the building data set of 351 vectors were divided into two groups at the ratio of 70:30, i.e., the learning data set (243 cases) used to construct the model, and the testing data set (108 cases) used to verify the model during the process of network training. The prediction capability of the developed models was evaluated with the use of a validation data set (117 cases) recorded in four independent storage experiments, performed under conditions falling within the scope of the main experiments (Table 4) and not included in model designing. It is worth emphasizing that there is no one universally accepted method for dividing experimental data into learning, test, and validation subsets that could provide a highly efficient network model. Nevertheless, it is known that too small a size of the validation set can make the verification of the quality of the obtained network unreliable [66]. In most cases, data set split percentages include: 50–80% of the parent base for learning, 15–25% for testing and 10–25% for validation [38,67,68,69]. In the study, individual sets (learning, test and validation) accounted for 52, 23 and 25% of the total data set (468 points), respectively.

#### 3.2.2. Artificial Neural Network Model Development

Artificial neural networks (ANNs) are a promising alternative to traditional mathematical modeling techniques, especially in the description of nonlinear phenomena that have not been precisely explained. Their undoubted advantages are connected with the ability to learn through experience, their internal self-adjustment to often complex relationships between input and output variables without the need to introduce a rigid model structure [70,71] and the capability to generalize the acquired knowledge. Another feature of neural networks, offering an extensive range of their applications, is connected with parallel processing of information by all units of the network structure, which greatly accelerates its action and, in many cases, facilitates signal processing in real-time. These characteristics give neural networks a certain superiority over other statistical methods used to solve prediction problems, particularly those associated with descriptions of nonlinear systems.

The theory of the aforementioned modeling technique is based on a simplified analogy to the human brain and its operating principle, based on the connections between individual elements of the network structure. These basic units of artificial neural networks, i.e., neurons (nodes), are arranged in a layered structure. The neurons of successive layers (input, hidden and output layers) are linked with each other through connections that are assigned weights. Moreover, the neurons of the hidden and output layers are equipped with activation (transfer) functions. The most common artificial neural networks are feed-forward neural networks, such as the multilayer perceptron (MLP) and radial basis function (RBF) networks, in which data pass the subsequent layers only from the input through hidden layer(s) to the output layer (without a feedback loop). In such networks, the input signal (data) passes through the neurons of successive layers, where it is transformed by the connection weights, biases and the activation functions of neurons in the hidden and output layers into the output variables. The construction of the neural network model consists in designing the network topology (the number of neurons in the input, hidden and output layers and the number of hidden layers) and determining the values of synaptic weights and biases, as well as selecting the type of transfer functions and estimating the value of their coefficients, so that specific input data leads to a specific target result.

In the study, the neural network models of phytosterol degradation were developed based on the multilayer perceptron (MLP) and radial basis function (RBF) networks. During the model design process, topologies with a single hidden layer containing from 2 to 16 neurons in the case of MLP-ANN, and from 2 to 50 in the case of RBF-ANN, were examined. The input layer contained three neurons corresponding to the independent variables (*a_w_*, *T*, *τ*), whilst the output layer contained one neuron corresponding to one dependent variable (phytosterol content). Additionally, different types of activation functions (linear (Lin), logistic (Log), exponential (Exp) and hyperbolic tangent (Than) for MLP and gaussian (Gau) for RBF) were tested in neurons of the hidden layer. Since previous studies showed that in output neurons of a regression-mode network, the linear function works best, the same type of function was applied in the research for both the MLP and RBF-based structures [25,45]. The applied feed-forward MLP-ANNs were trained with the Broyden–Fletcher–Goldfarb–Shanno (BFGS), whilst RBF-ANNs were with the Red Baron Flight Training (RBFT) learning algorithm, to estimate parameters of network models. The structure of the neural network models was determined by the trial and error method. The range of the number of neurons in the hidden layer of MLP-ANNs (*N_h_*) tested in the study was estimated using the formula that assumes five learning cases for each model coefficient controlling the calculations performed by the network (all synaptic weights and biases, i.e., *N_in_·N_h_* + *N_h_* + *N_out_·N_h_* + *N_out_*) as follows: *N_h_* = *(L-5*⋅*N_out_*)/(5⋅(*N_in_* + *1* + *N_out_*)), where *N_in_*—is the number of input nodes, and *L*—is the number of learning cases [25,42]. The aim of the training process is to minimize the error between the target output vector and output signals calculated by the ANN, hence the prediction performance of the developed ANN models was evaluated based on the errors calculated for the learning, testing (internal verification) and validating (external verification) data sets. For each tested topology, 1000 neural network models (in total 109.000 networks) were developed using the artificial neural network package from the Statistica 13.3 software (StatSoft, Tulsa, OK, USA).

#### 3.2.3. Response Surface Regression Modeling

Apart from the ANN technique, a second-order-response surface regression (RSR) was also used to mathematically describe the kinetics of phytosterol degradation in a stored bulk of rapeseeds as a function of temperature, water activity in seeds and time. The RSR model was formulated by a least-square fitting of a second-order surface response regression equation to the experimental data (building data set). This methodology combines features of both polynomial regressions and factor regression with the effect of the two-way interaction of the predictor variables. The general equation of the hierarchical model for a quadratic response surface regression containing three predictors took the following form:

(2) $$y=\beta_{0}+\overset{k}{\underset{i=1}{\sum }}\beta_{i}\cdot x_{i}+\overset{k}{\underset{i=1}{\sum }}\beta_{ii}\cdot x_{i}^{2}+\underset{i<j}{\sum }\sum \beta_{ij}\cdot x_{i}\cdot x_{j}+\varepsilon$$

where: *y* is the dependent variable; *β***_0_** is the intercept; *β*_1_, …, *β*_k_ are regression coefficients; *x_1_*, …, *x_k_* are predictors (*a_w_*, *T* and *τ*), *ε* is the standard estimation error. The coefficients of the equation were determined using the response surface regression analysis in a package from the Statistica 13.3 software (StatSoft, Tulsa, OK, USA) based on the building data set. The validation data set was used for the final verification of the formulated model.

#### 3.2.4. Model Performance Assessment

The capability of the formulated models was evaluated with the use of the Statistica 13.3 software (StatSoft, Tulsa, OK, USA) at a significance level of α = 0.05. The fit of the model response to the experimental data was assessed on the basis of the explained variation (*R*^2^), mean absolute error (*MAE*) and the root mean square error (*RMSE*) expressed with the following expressions:

(3) $$MAE=\frac{1}{n}\cdot \sum \left|PS_{E}-PS_{M}\right|$$

(4) $$RMSE=\sqrt{\frac{\sum {\left(PS_{E}-PS_{M}\right)}^{2}}{n}}$$

where *n*, the number of experimental points; *PS_E_*, experimental total phytosterol level; *PS_M_*, estimated phytosterol level. A bias factor (*B_f_*) and a mean relative percentage error (*MRPE*, %) used to determine the mean deviation of predicted values from experimental ones were calculated as follows:

(5) $$B_{f}=10^{\left(\sum \log\left(\frac{PS_{M}}{PS_{E}}\right)/n\right)}$$

(6) $$MRPE=\frac{1}{n}\cdot \sum \frac{PS_{E}-PS_{M}}{PS_{E}}\cdot 100$$

The overall prediction error and the distance between predicted and experimental values were expressed by the accuracy factor (*A_f_*) and the mean absolute relative percentage error (*MAPE*, %) calculated as follows:

(7) $$A_{f}=10^{\left(\sum \left|\log\left(\frac{PS_{M}}{PS_{E}}\right)\right|/n\right)}$$

(8) $$MAPE=\frac{1}{n}\cdot \sum \left|\frac{PS_{E}-PS_{M}}{PS_{E}}\right|\cdot 100$$

Finally, in addition to the statistical indicators, the elliptical joint confidence region (EJCR) test was performed to assess the correlation linearity between the experimental data and the model predictions according to the methodology described by González [72] and Olivieri [59]. The intercept and the slope of the correlation curve determined for each model by the regression analysis were compared with the theoretically ideal values (0, 1). The elliptical region was determined by taking the critical F values for the Snedecor–Fisher statistic at a 95% confidence level. The EJCR methodology assumed that the points being inside the elliptical region are consistent with the observed data at the chosen confidence interval. If the ideal point (1, 0) is included in the elliptical joint confidence region, it can be assumed that there is no bias between the prediction and measurement [72].

## 4. Conclusions

Previous research has shown that phytosterols can influence cholesterol metabolism; nevertheless, their concentration in the natural human diet is often too low to effectively reduce serum cholesterol; therefore, there is a growing demand for products enriched with these components. Rapeseed oil is the main source of phytosterols; however, due to the lability of these particles, their considerable amounts may be lost during postharvest seed treatment. Taking this into account, the prediction of the dynamics of phytosterol degradation is crucial to optimize storage processes and identify conditions leading to the reduction in their content. Up to now, there are no concepts for predicting tools to simulate changes in phytosterol concentration in a mass of stored rapeseeds, therefore, to fill the gap in the current state of knowledge, in this study, mathematical models based on artificial neural networks and response surface regression were developed. The obtained results revealed the applicability of elaborated models to predict phytosterol degradation in a stored bulk of rapeseed in time as a function of seed temperature and water activity, which are readily measurable in practice parameters. The accuracy of the developed models depended on the modeling technique, and in the case of ANN, also on the network topology, and it was as follows: MLP-ANN > RBF-ANN > RSR. The ANN-based modeling technique turned out to be more effective in estimating the levels of mentioned bioactive components than the response surface regression. Among the tested types of networks, the MLP-ANNs proved to be more satisfactory in the prediction of examined component concentrations than RBF-ANNs. The MLP-ANN network, characterized by the best efficiency, was selected as the model to predict phytosterol degradation. This network had a fairly compacted topology contained in a hidden layer of 9 neurons equipped with a Log activation function. The evaluation of the developed MLP-ANN performance indicates that the elaborated model can be successfully used as a support management tool in postharvest seed preservation and storage systems.

## Figures and Tables

**Table 1:** Basic information on the structure and learning, test and validation error values of MLP and RBF neural networks selected as models of the neural network to predict phytosterol degradation in a stored bulk of rapeseed.

Network Parameters	Artificial Neural Network	
	MLP 3-9-1	RBF 3-27-1
Number of observation points (total)	468	
Learning	243	
Test	108	
Validation	117	
Activation functions in hidden layer	Log	Gau
Activation functions in output layer	Lin	Lin
Learning error	0.0047	0.0147
Test error	0.0074	0.0153
Validation error	0.0098	0.0213
Learning accuracy	0.9969	0.9903
Test accuracy	0.9944	0.9886
Validation accuracy	0.9887	0.9769

**Table 2:** Results of the response surface regression: regression coefficients, standard errors and probability levels for the developed regression model.

Equation Variable and Intercept	Regression Coefficients	Standard Error	*p*-Values
Intercept	−55.1677	5.6557	<0.0001
*T*	0.9326	0.0438	<0.0001
*T* ^2^	−0.0037	0.0005	<0.0001
*a_w_*	132.8619	13.3651	<0.0001
*a_w_* ^2^	−69.4989	7.9210	<0.0001
*τ*	0.2277	0.0163	<0.0001
*τ* ^2^	0.0001	0.0001	0.0129
*a_w_* × *T*	−1.0046	0.0464	<0.0001
*T* × *τ*	−0.0013	0.0001	<0.0001
*a_w_* × *τ*	−0.2870	0.0176	<0.0001

**Table 3:** Values of indicators used to evaluate performance of ANN and RSR models to predict the content of phytosterols in a stored bulk of rapeseed calculated for all data sets: L—learning, T—test B—building (L + T) and V—validation.

Statistical Index	Model							
	MLP-ANN			RBF-ANN			RSR	
Data set	B		V	B		V	B	V
	L	T		L	T			
Coefficient of determination (*R*^2^)	0.994	0.989	0.978	0.981	0.977	0.954	0.930	0.933
Root mean square error (*RMSE*)	0.097	0.122	0.140	0.172	0.175	0.206	0.320	0.302
Mean absolute error (*MAE*)	0.080	0.099	0.117	0.128	0.143	0.148	0.242	0.248
Mean relative percentage error (*MRPE*, %)	−0.032	0.209	−0.653	−0.063	0.084	−1.224	−0.378	−3.477
Bias (*B_f_*)	1.000	0.998	1.006	1.000	0.999	1.012	1.002	1.034
Mean absolute relative percentage error (*MAPE*, %)	1.384	1.658	1.922	2.270	2.486	2.516	4.215	4.121
Accuracy factor (*A_f_*)	1.014	1.017	1.019	1.023	1.025	1.025	1.043	1.041

**Table 4:** Storage conditions: temperature and water activity in rapeseeds applied in experiments used for model development and validation and the type of experiment indicating the purpose of data use: B—building and V—validation.

Storage Conditions																
Temperature (*T*)	*T* = 12 °C				*T* = 18 °C				*T* = 24 °C				*T* = 30 °C			
Water activity (*a_w_*)	0.76	0.80	0.86	0.90	0.76	0.80	0.86	0.90	0.75	0.81	0.85	0.90	0.75	0.80	0.84	0.90
Experiment type	B	B	V	B	B	V	B	B	B	B	V	B	B	V	B	B

## Data Availability

All data are available from the corresponding author upon request.

## Sample Availability

Samples of the compounds are not available from the authors.
